# Foundational principles for young intensivists to drive better outcomes: palliative care

**DOI:** 10.62675/2965-2774.20260301

**Published:** 2026-04-24

**Authors:** Daniel Neves Forte, Edison Iglesias de Oliveira Vidal, Nancy Kentish-Barnes

**Affiliations:** 1 Universidade de São Paulo Faculdade de Medicina Emergency Department, Central Institute, Hospital das Clínicas São Paulo SP Brazil Emergency Department, Central Institute, Hospital das Clínicas, Faculdade de Medicina, Universidade de São Paulo - São Paulo (SP), Brazil.; 2 Universidade Estadual Paulista "Júlio de Mesquita Filho" Faculdade de Medicina Geriatrics Division, Internal Medicine Department Botucatu SP Brazil Geriatrics Division, Internal Medicine Department, Faculdade de Medicina, Universidade Estadual Paulista "Júlio de Mesquita Filho" - Botucatu (SP), Brazil,; 3 Saint Louis University Hospital Medical Intensive Care Unit, Famiréa Research Group Paris France Medical Intensive Care Unit, Famiréa Research Group, Saint Louis University Hospital - Paris, France.

As a young intensivist in the early 2000s, one of the authors was intensely focused on developing competencies to manage refractory shock, appropriately indicate dialysis, and perform other technically challenging procedures in the intensive care unit (ICU). At the time, this emphasis on the patient's biological dimension led to a lived experience of inattentional blindness^(1)^ - a failure to recognize other essential aspects of patient care. This may have been exacerbated by prevailing assumptions: that symptom control is less important than disease control; that communication with families depends on goodwill or innate talent; or that teamwork naturally emerges from clinical expertise alone. This narrow approach resulted in a growing sense of ineffectiveness, along with the perception that conflict was ubiquitous and driven by others. Ultimately, this led to burnout.

In 2005, a turning point occurred when this intensivist was first introduced to the idea that communication and end-of-life care could be approached with method and evidence. This marked the beginning of an engagement with a broader field of knowledge: palliative care (PC). Now, nearly 20 years later, what was once unseen has become clear: suffering -the suffering of patients, families, the clinical team, and ourselves. Crucially, the ability to care for suffering requires a distinct set of competencies from those used to treat disease. These skills are not intuitive or based solely on personality; they are evidence-based and require study, training, and practice - just like any other clinical discipline. And they can be integrated into the intensivist's daily practice.

The concept of PC has evolved from a focus on end-of-life care for terminally ill patients to a broader approach aimed at relieving suffering and improving quality of life for people - and their families and caregivers - experiencing profound health-related suffering due to severe illness.^(2)^ A noteworthy part of the evolution involved abandoning the outdated dichotomy of curative care *versus* PC. Throughout this evolution, attention to physical, psychological, social, and spiritual aspects of suffering, along with a strong emphasis on interprofessional collaboration, has remained central to how PC is understood and delivered.

More than just a clinical approach or a set of techniques, PC should be understood as a philosophy of care that was developed in response to the sheer failure of the medical field to relieve the suffering of people with severe illnesses, particularly at the end of life.^(3)^ If medicine and the healthcare field had accomplished a better job at treating pain, relieving other uncomfortable symptoms, communicating effectively, and engaging in shared decision-making to mitigate the suffering of patients with serious illnesses, their families, and caregivers throughout history, PC would never have emerged.

Despite progress, there is consistent evidence that highlights the challenges faced by ICUs worldwide in adequately addressing the suffering of critically ill patients and their loved ones. For example, a recent review reported prevalence rates of uncontrolled pain, breathlessness, and thirst in ICU patients ranging between 40 and 77%, 33 and 44%, and 30 to 70%, respectively.^(4)^ Other reviews have shown that communication between healthcare professionals and patients/family members is often suboptimal (e.g., unclear, incomplete, untimely, lacking cultural competency/humility, and exploration of values and preferences of care), contributing to distress, dissatisfaction, and conflict.^(2)^ These issues have been associated with long-term suffering, as in several cases of post-ICU syndrome.^(5)^

At the same time, a growing body of evidence supports integrating PC into intensivists’ daily practice, demonstrating improvements in ICU care. Examples include addressing the thirst of intubated patients,^(6)^ managing dyspnea, while simultaneously treating, when possible, respiratory failure,^(7)^ and understanding opioid conversion, such as between morphine and fentanyl, to prevent excessive dosing. Moreover, communication is increasingly recognized as a clinical procedure, requiring structured methods for engaging with families and a coordinated team approach throughout their ICU stay.^(8,9)^ These interventions yield measurable and meaningful outcomes, not only for patients and their families, but also for healthcare teams and the broader health system. A recent systematic review including 9 randomized controlled trials and 49 cohort studies acknowledged methodological limitations such as small sample sizes, single-center studies, and heterogeneity in interventions, but also identified encouraging trends.^(10)^ Notably, 11 studies reported a reduction in length of stay, 5 demonstrated lower healthcare costs, and 2 described decreased mortality rates.

Recognizing both the ethical imperative and the growing evidence base, several international societies have recommended integrating PC into standard ICU practice.^(11–14)^ Indeed, PC is not an "add-on" but a fundamental component of good intensive care medicine. When integrated early and systematically, PC can become a standard of care that complements efforts to optimize patient outcomes. It invites a broader view of the intensivist's role, not only in managing physiology but also in supporting families, guiding care through uncertainty, and helping ensure respect for patients’ values.^(11)^ Clinical care and PC knowledge and skills can be metaphorically envisioned as two hands (Figure 1). Using them in a coordinated manner will often lead to less distress and better outcomes than when applied in isolation.

**Figure 1:**
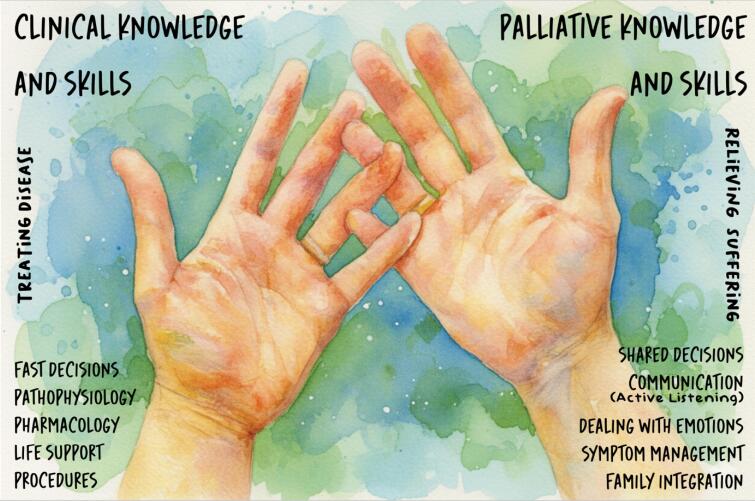
The two-hands metaphor: clinical and palliative care knowledge and skills are like two hands; when used together in a coordinated way, they lead to less distress and better outcomes than when used in isolation.

Image generated with artificial intelligence (both Microsoft Copilot^TM^ and Gemini^TM^) based on prompts and a photograph by Fernanda Bono Fukushima, with further editing by the artist.

To achieve this and address the complexity of critical care, doctors and other ICU clinicians must be prepared not only technically but also ethically, emotionally, and relationally.^(15,16)^ Indeed, research has shown that end-of-life in the ICU, as well as communication problems, are associated with increased risk of clinician burnout.^(17)^ Interestingly, PC offers a framework to ICU clinicians to better care not only for patients and families, but also for themselves. By fostering shared decision-making with patients, families, and colleagues, PC helps move towards a model based on partnership, humility, and trust. This collaborative approach can lessen clinician burnout by sharing decision-making responsibility, reducing moral distress, and strengthening team support, while also providing clinicians with assurance that the decisions made are meaningful.

Importantly, the principles of PC are not only relevant when death is near. They strengthen the care delivered by ICU clinicians in all situations, even when a cure seems possible. Furthermore, they can even improve survival^(10)^ and be biologically based.^(18)^ These principles insist that intensive care is about more than survival: it is also about quality of life during and, when possible, after the ICU stay. Indeed, improving communication to elicit patients’ needs and concerns - including at the end of life - can lead to more patient-centered, responsive care.^(19)^

None of this can be achieved alone. Interdisciplinary collaboration is essential: integrating the perspectives of nurses, social workers, or psychologists allows us to see the patient as a person, not just a clinical problem.^(20)^ This collaborative spirit must also extend to medical training and research. Young doctors play a crucial role in advocating for the formal inclusion of PC principles in ICU curricula and in contributing to the development of high-quality research that defines best practices. Providing incentives - such as protected research time, mentorship programs, and formal acknowledgment of PC work in professional promotion - can encourage engagement and help build a robust evidence base.

Returning to the author's experience presented at the beginning of the paper, today, almost 20 years later, it has become clearer what was missing for that young intensivist. Integrating PC into routine intensive care not only enhances effectiveness and patient outcomes but also fosters resilience, meaning, and the joy we can experience in our profession as human beings. It is not about doing less, it is about doing better.

## Data Availability

All the contents underlying the manuscript text are already available in their entirety and without restrictions.
